# Recent Advances in the Molecular and Cellular Mechanisms of gp120-Mediated Neurotoxicity

**DOI:** 10.3390/cells11101599

**Published:** 2022-05-10

**Authors:** Valeria Avdoshina, Italo Mocchetti

**Affiliations:** Laboratory of Preclinical Neurobiology, Department of Neuroscience, Georgetown University Medical Center, Washington, DC 20057, USA; va44@georgetown.edu

**Keywords:** axonal damage, HIV, HAND, neurotrophins, neuronal cytoskeleton, p75NTR, tubulin beta 3, viral proteins

## Abstract

Axonal degeneration and loss of synapses are often seen in different brain areas of people living with human immunodeficiency virus (HIV). Nevertheless, the underlying causes of the pathological alterations observed in these individuals are poorly comprehended, considering that HIV does not infect neurons. Experimental data have shown that viral proteins, including the envelope protein gp120, cause synaptic pathology followed by neuronal cell death. These neurotoxic effects on synapses could be the result of a variety of mechanisms that decrease synaptic plasticity. In this paper, we will briefly present new emerging concepts connected with the ability of gp120 to promote the degeneration of synapses by either directly damaging the axonal cytoskeleton and/or the indirect activation of the p75 neurotrophin receptor death domain in dendrites.

## 1. Introduction

The term human immunodeficiency virus type 1 (HIV)-associated neurocognitive disorder (HAND) has been used to describe a variety of neurocognitive dysfunctions in people living with HIV (PLWH). This condition, especially the milder form, can be seen in almost 50% of PLWH despite the introduction of combination antiretroviral therapy (cART) [1]. The failure of cART to reduce HAND can be multifactorial and may include inefficient penetration of drugs through the blood–brain barrier (BBB) as well as ongoing viral replication due to drug resistance. Each phenomenon results in an ineffective elimination of the virus in the central nervous system (CNS). Moreover, it appears that HIV reduces the innate capability of the CNS to adjust to injury. Thus, the need to develop a cure for HAND is of paramount importance and remains a great challenge in the neuroscience community.

HAND comprises several different types of cognitive impairments, predominantly presenting with deficiencies in attention, memory, processing speed and executive function. Emotional manifestations, such as depression and apathy, and, to a lesser degree, motor deficits also occur [2]. Impairments of memory and executive functioning often affect workplace functioning and medication adherence, as does depression. Risk factors that could explain how HAND develops include lower education, severe pre-existing immunosuppressive conditions, older age, history of AIDS-defining illnesses, high plasma levels of pro-inflammatory cytokines and lower CD4 count. However, as for other neurodegenerative diseases, multiple neurotoxic events could be in place to reduce the natural ability of the CNS to cope with neuronal degeneration. The decision of a neuron to “die” is a physiological program that is operative only during CNS development and requires an active process. Often, during development, neurons destined to die are pruned. What is remarkable is that HIV infection of the CNS appears to trigger the intrinsic mechanisms of neuronal “pruning”, studies having shown a decrease in synaptic density in HAND brains [3,4]. However, the molecular basis for this pathology remains to be elucidated. The knowledge of the mechanisms leading to synaptic loss may be critical because the optimal treatment may depend on the state of the CNS at risk, whether neuroinflammation or other cellular events may be present. Clearly, advances in the clinical therapy of HAND will depend on knowledge gained from the multiple experimental systems that are presented in this paper.

## 2. HAND Pathology and Viral Proteins

### 2.1. HAND

Very early after seroconversion, HIV penetrates the blood–brain barrier (BBB) and enters the brain parenchyma via infected lymphocytes and monocytes and probably by transependymal migration [5]. Once inside the brain, CNS immunocompetent cells such as microglia become the primary source of infection [6]. Perivascular astrocytes are also infected [7]. Neurons and oligodendroglia are not or are only minimally infected. Although brain infection occurs early in the days after primary infection, the development of HAND takes years. As an explanation for this ostensible contradiction, it has been suggested that, initially, the brain infection is relatively well controlled, while later there is a quantitative and qualitative breakdown of immune control in the CNS.

HAND is currently sub-classified into three forms, according to the Frascati criteria [8]: asymptomatic neurocognitive impairment (ANI), mild neurocognitive disorder (MND) and HIV-associated dementia (HAD). ANI is currently the most prevalent manifestation of HAND. Individuals with ANI usually have mild cognitive impairment, demonstrated by formal neuropsychological tests, without any observed abnormality in everyday functioning. Patients with MND exhibit varying degrees of cognitive impairment that interfere with activities of daily living. Although the impairment does not meet criteria for delirium or dementia, it is a mild-to-moderate impairment of cognitive function, not nearly as dramatic as HAD, the most severe form of HIV dementia. Pathologically, HAND is characterized by HIV encephalitis (HIVE) and HIV leukoencephalopathy. The encephalitis is an inflammatory response characterized by disseminated infiltrates of lymphocytes, macrophages and multinucleated giant cells, while leukoencephalopathy implies bilateral diffuse loss of myelin in the hemispheric white matter along with astrocytosis and microglial infiltration. However, HIVE has become less common in HAND after the introduction of cART and is not a necessary feature of all HAND pathogenesis [4]. In fact, HIVE is rarely seen in ANI and/or MND [9]. Thus, HAND cannot be a consequence of HIVE only.

### 2.2. Neuroinflammation

Our understanding of how HIV damages CNS neurons is incomplete. This is most likely because, as for other neurodegenerative diseases, the underlying molecular and cellular mechanisms promoting pathogenesis remain poorly understood. Neurons are not infected but are particularly sensitive to the neurotoxic action of HIV as demonstrated by the loss and dysfunction of synapses, as well as by neuronal degeneration in HAND. HAND could be caused by a complex interplay among the virus, the immune response and glial cells. This is in line with the current view that the CNS-resident immune cells (microglia, astrocytes and oligodendrocytes) which serve supportive and nutritive roles for neurons [10] can also engage from time to time in several “inflammatory” processes. Indeed, activated glial cells produce pro-inflammatory cytokines and other neurotoxins, including reactive oxygen species [11]. While some of these inflammatory processes defend the CNS from pathogens and help it to recover from stress and injury, a prolonged activation of glial cells can result in a more severe and chronic neuroinflammatory cycle that actually promotes or propagates neurodegeneration. However, activated microglia do not necessarily correlate or predict neurocognitive impairment. Thus, neuronal degeneration in HAND could be the result of indirect neurotoxicity mediated by glial cell activation which culminates in the excessive release of inflammatory cytokines, metabolites and viral proteins from virally infected non-neuronal cells. On the other hand, viral proteins can be directly neurotoxic through various mechanisms, as demonstrated experimentally by in vitro and in vivo findings, as detailed in the next section. Thus, HAND is not caused by a one-dimensional and direct pathogenetic event but rather by multi-dimensional and complex immunopathological processes that are governed by viral as well as host factors.

### 2.3. Viral Proteins

The HIV genome codes for both regulatory and structural proteins (reviewed in [12]). Regulatory proteins include the transactivator of transcription (Tat) and the RNA splicing-regulator (Rev), which are necessary for the initiation of viral replication. Additional regulatory proteins play important roles in efficient viral replication and budding. These include the negative regulating factor (Nef), the viral protein R (Vpr), the viral protein U (Vpu) and the viral infectivity factor (Vif).

Structural proteins include the Gag (group specific antigen) polyprotein and Pol polyprotein, which are responsible for a variety of functions, including the formation of the structural components of HIV, the reverse transcription of viral enzymes and the proteolytic cleavage of important proteins and molecules. Adjacent to the *pol* sequence, there is the *env* gene, which codes for the two envelope glycoproteins gp120 and gp41. These two proteins are important for the binding of HIV to the host receptor CD4.

Among all these proteins, gp120 [13], gp41 [14], vpr [15], Tat [16], rev [17] and nef [18] have been shown to promote neurotoxicity. Neurotoxicity is a term used broadly to indicate the inability of the CNS to oppose various events prompted by HIV initiating neuronal damage. Viral proteins are produced and released from infected cells. These cells in the CNS include resident immune cells, such as microglia, as well as non-resident macrophages [19,20]. In addition, perivascular astrocytes, which have been suggested to act as HIV reservoirs, can produce low levels of HIV virions when reactivated [21].

**Figure 1:**
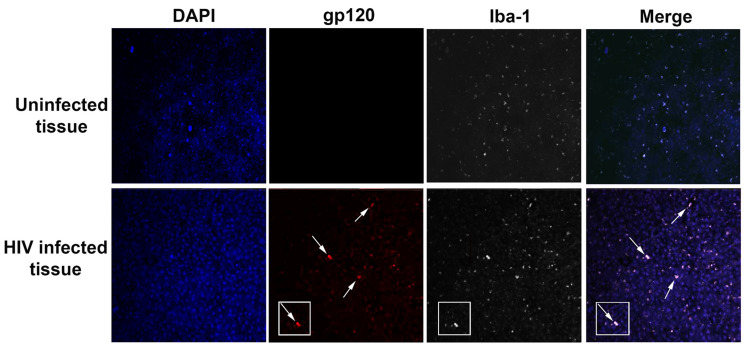
Detection of gp120 proteins in the cortex of MND. Examples of cortical sections from uninfected and HIV-infected individuals with MND (samples are from NNTC, viral load < 40 copies/mL). Sections were deparaffinized, stained for nuclei (DAPI), gp120 biotinylated antibody (Cy3, NIH AIDS Reagent Program) and Iba-1 (Cy5), and analyzed by confocal microscopy using a Nikon A1R microscope and NIS elements. Arrows and insets show that few microglial cells are positive for gp120 and are only detected in MND. Data were reproduced in two additional cases. (In collaboration with Dr. E. Eugenin, UTMB, Galveston, TX, USA.)

Viral proteins are either “shed” from the virus during the initial phase of infection or actively released from infected cells, as in the case of Tat and gp120 [22]. The envelope protein can also be released when the virus is incorporated into host cells [23]. These proteins become available to surrounding cells and can be either degraded in the extracellular space or be endocytosed through low-density lipoproteins [24] or a chemokine receptor-mediated mechanism [25]. Although detection of viral proteins remains a challenge, studies have shown that HAND subjects have detectable levels of these proteins in their brains [26,27,28] or CSF [29], although their expression could be limited to a very few microglia cells, as in the case of gp120 (Figure 1).

**Figure 2:**
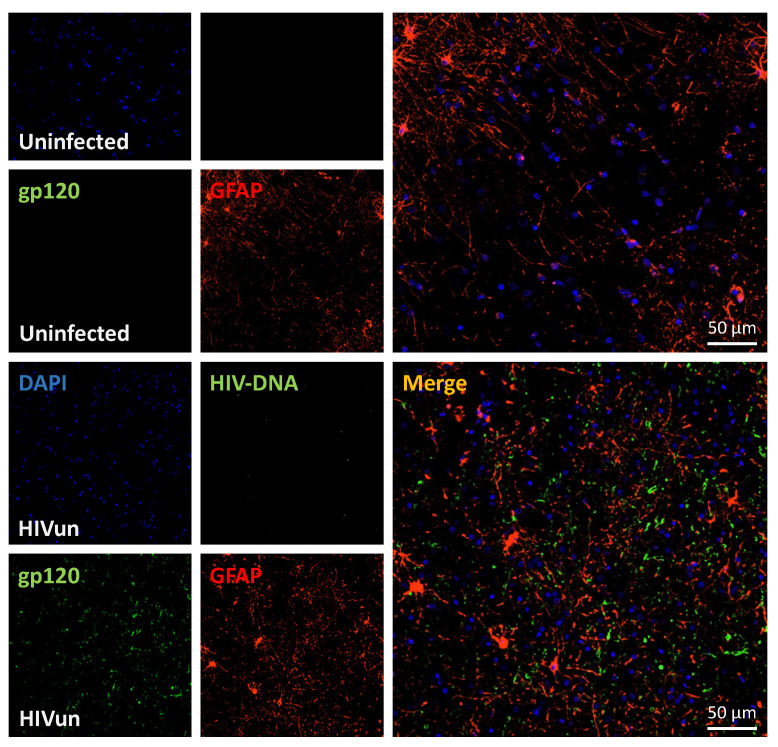
HIV-gp120 protein is not expressed in astrocytes but in surrounding cells. Sections were prepared as described in Figure 1. Representative confocal images showing DAPI staining, HIV DNA, astrocytes (GPAF) and gp120 protein in uninfected and HIV-infected cerebral cortexes. Merge shows that gp120 is mostly expressed in cells surrounding astrocytes in the HIV-infected cerebral cortex. (In collaboration with Dr. E. Eugenin, UTMB, Galveston, TX, USA.)

The envelope protein can also be found in cells surrounding astrocytes (Figure 2). Moreover, infected non-CNS cells, such as pericytes [30], choroid plexus [31] and macrophages, can release neurotoxic viral proteins, such as Tat and gp120, which cross the blood–brain barrier by various mechanisms [32,33].

## 3. gp120 Neurotoxicity

gp120 is the envelope protein that binds to chemokine co-receptors CCR5 and CXCR4 and allows the virus to change conformation and enter cells. This binding occurs through a specific sequence within gp120 termed the V3 loop [34]. Co-receptor binding induces gp41 fusion peptide insertion into the host cell membrane, resulting in membrane fusion. Following the fusion reaction, cytoskeletal rearrangements facilitate release of the viral core into the cytosol. CCR5 or CXCR4 expression on target cells determines which strain of HIV will infect these target cells. Thus, gp120 binding to CCR5 or R5 will primarily promote the infection of macrophages and microglia [35], and therefore these strains of HIV are also called M-tropic viruses. CXCR4 is expressed by T lymphocytes and mediates infection of T-tropic virus, which is referred to as X4. Dual tropism viruses that use both co-receptors are designated R5x4. All these strains have been found in the brain [36,37,38]. Antagonists of CCR5 or CXCR4 receptors prevent HIV entry into CD4 cells and have been used to block the spread of infection [39,40]. Moreover, humans with a nonfunctional CCR5 because of a Δ32 mutation are resistant to HIV-1 infection [41].

### 3.1. Co-Receptors in the CNS

CCR5 and CXCR4 are expressed by various cells in the mature CNS [42]. Therefore, several studies have been undertaken to characterize the role of gp120 binding to CXCR4 or CCR5 in the pathogenesis of HAND. While neurons did not appear to be the main target of the virus, since the widespread neuronal damage is not associated with a productive viral infection in neurons, studies support the hypothesis that an indirect mechanism exists to explain the neuronal disfunction which occurs in PLWH. Specifically, it has been shown that gp120 affects neuronal survival through a direct interaction with chemokine receptors expressed by neurons and/or with non-neuronal cell types, such as monocytes, macrophages/microglia and astrocytes [43]. How the activation of chemokine receptors causes neurodegeneration is still under investigation. Both CXCR4 and CCR5 trigger multiple signaling pathways upon binding to their physiological ligands. Known ligands for CXCR4 are stromal cell-derived factor 1 (SDF-1) or CXCL12, which binds only to CXCR4, and macrophage migrating inhibitor factor [44], a pro-inflammatory cytokine that also binds to other receptors. CXCR4 and CXCL12 are crucial for CNS development and in particular for promoting hippocampal neurogenesis [45]. Thus, activation of CXCR4 signaling, at least during CNS development, appears to have proliferative rather than neurodegenerative effects.

The preferred ligand for CCR5 is CCL5, a chemokine that is proinflammatory in immune cells but which also exhibits regenerative [46] properties in the brain. Moreover, CCL5 has been shown to be neuroprotective against HIV [47]. Activation of CCR5 is neuroprotective by promoting dephosphorylation of the somatodendritic voltage gated K^+^ channel Kv2.1 [48]. However, the removal of CCR5 in mice leads to recovery of motor function after stroke [49]. In addition, CCL5 is neuroprotective when it activates a non-chemokine receptor named GPR75 [50]. Thus, it appears that CCL5 is neuroprotective depending on the type of receptor it activates.

Chemokine receptor signaling could be neurotoxic or neuroprotective depending upon the cell types that express them. For instance, in microglia and/or macrophages in vitro, gp120 promote the secretion of neurotoxins, such as quinolinic acid, arachidonic acid, free radicals, cytokines (TNF-α, IL1-β, IL-6), chemokines and other as yet unidentified factors [51,52,53,54]. In astrocytes, gp120 alters the exchange of Na^+^/H^+^ and glutamate efflux [55]. Astrocytes are also activated by gp120 to produce inflammatory cytokines, such as TNF-α, [56], IL-1β and IL-6 [57], and constitutive nitric oxide synthase [58]. The production of inflammatory cytokines leads to gp120-induced alterations in Ca^2+^ dynamics, ATP release and the opening of conenxin-43 hemichannels, which could result in degeneration of neuronal processes [59]. A major caveat of gp120 research, as for other viral proteins [60,61], is whether the concentrations of gp120 used experimentally may exceed those found in HAND. Likewise, the release of pro-inflammatory/neurotoxic compounds in vitro is often the hallmark of an acute phenomenon, whereas HAND is a chronic disease and its clinical manifestation may take several years.

The theory that HAND could be mediated by secreted neurotoxins and/or inflammation is supported in human studies using position tomography [62] or data showing elevated serum levels of proinflammatory cytokines in cerebrospinal fluid from HAND patients [63]. However, these data do not necessarily correlate the amount of neurotoxicity with the degree of cognitive impairment. Moreover, there is no clear clinical evidence showing that an infected person with neuroinflammation will ultimately develop HAND.

### 3.2. Animal Models of HAND

Neuroinflammation due to persistent viral presence in the CNS may partly provide explanations of HAND pathology [64,65]. However, HIV infection has been shown to lead to metabolic comorbidities, including atherosclerosis, diabetes, and impaired cholesterol metabolism [66]. Moreover, several viral proteins, including gp120, have been shown to promote neurotoxicity even without causing neuroinflammation. Thus, additional mechanisms underlying HAND should be considered. Many studies have focused on the neurotoxic effects coordinated by HIV proteins and their molecular and cellular mechanisms that lead to direct neuronal loss [67,68]. As the basic mechanisms activated by HIV proteins become better understood, novel strategies can be devised to prevent their neurotoxicity.

One of the major obstacles in testing and discovering mechanisms of neurotoxicity for HAND is the availability of animal models that recapitulate all symptoms of HAND. Simian immunodeficiency virus infection of nonhuman primates is a useful model for testing vaccines and finding a cure for HIV [69]. However, this model is not easily accessible to most researchers. Several laboratories have independently confirmed in rodent models of HAND the presence of synaptic loss, similar to what is seen in postmortem brain tissue from HAND subjects. For example, HIV transgenic rats generated with an integrated HIV genome [70] exhibited a deficit in the synaptic marker debrin [71] and abnormal mitochondrial function [72]. This aberrant pathology occured even in the absence of microglial activation [73,74]. Humanized NOD-SCID mice infected with HIV also showed a decrease in synaptic markers [75] as well as impaired oligodendrocyte differentiation [76]. This mice were generated by intrahepatic transplantation of human CD34+ cells from fetal liver tissue into immunodeficient CD17-scid (SCID) micelacking mature B and T cells [77,78]. A similar scenario has been observed in mice over-expressing gp120 under a glial fibrillary acidic protein promoter (gp120tg). Their synaptic simplification includes loss of dendritic processes and a decrease in synaptic density in various brain areas and decreased long-term potentiation [13,79]. Similarly, exposure of rodent neurons in vitro to nanomolar concentrations of gp120 promotes loss of neuronal processes and apoptosis [80,81,82,83]. Thus, animal models can reproduce the dendritic injury seen in HAND and can be used, at least experimentally, to gain insights into the causes of biological processes that underlie the development of neurodegeneration.

## 4. gp120 and Neuronal Toxicity: Beyond the Inflammation Theory

### 4.1. gp120 Is Endocytosed into Neurons

**Figure 3:**
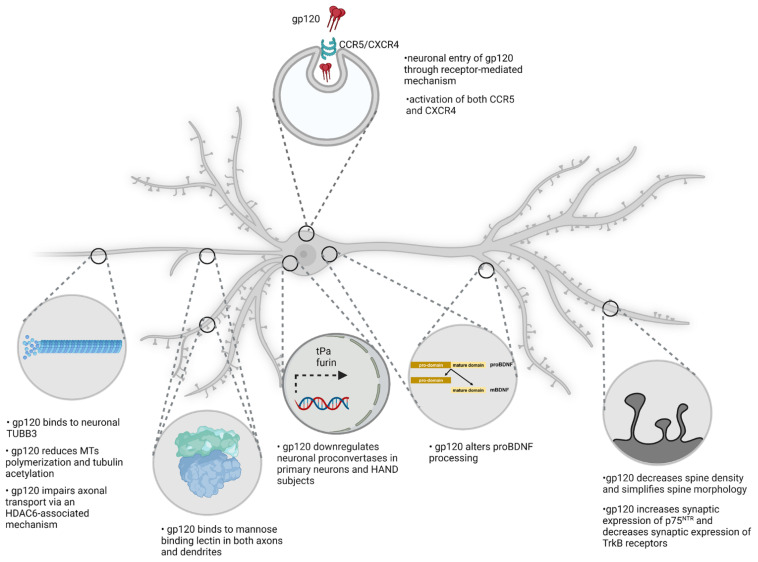
Proposed mechanism of gp120-mediated decrease in spine density. gp120 binds and activates co-receptors CXCR4 or CCR5. These receptors are critical for gp120 endocytosis. Once inside neurons, gp120 binds to microtubules and changes the post-translational modification of tubulin, which could result in axonal damage. Internalized gp120 is also transported by axons or dendrites back to the Golgi apparatus via a mannose-binding lectin. In the Golgi apparatus, gp120 down-regulates the activity of furin, which results in decreased processing of proBDNF to mature BDNF. When proBDNF is released, it binds to p75NTR and causes a loss of dendritic spines.

HIV does not infect neurons; nevertheless, it produces neuronal damage and synaptic loss when added to neurons in culture [83]. Similar to HIV, neurons exposed to gp120 exhibit axonal swelling. When swelling becomes severe, it forms a bulb along the site of the break in the cytoskeleton. The bulb is a tear of the axon caused by a drawing back toward the cell body. The neurotoxic effect of HIV is blocked by co-receptor antagonists or gp120 inhibitors [83], supporting the notion that HIV “sheds” gp120, which then acts independently from the virus and promotes neurotoxicity by binding to these chemokine receptors [82,84]. Moreover, it was found that gp120 can be endocytosed in neurons [25]. Intracellular gp120 (Figure 3) binds to mannose-binding lectin [85], a carrier that facilitates glycoprotein trafficking from the endoplasmic reticulum to the Golgi apparatus [86]. A mannose-binding lectin–gp120 complex associates with subcellular vesicles that traffic along neurites and could carry gp120 toward the soma [85]. This suggestion is consistent with results showing that gp120 is retrogradely transported from the synaptic cleft to the perinuclear region of neurons [87,88]. The transport is mainly axonal because both colchicine and nocodazole, two agents that bind to tubulin and prevent its addition to growing microtubule ends, block this event [85,88]. Interestingly, only a small amount of gp120 is seen inside lysosomes [88] or associated with lysosome-associated membrane glycoprotein 2-positive organelles [87], suggesting that gp120 is not efficiently degraded by the endogenous autophagic process. Therefore, it is plausible to suggest that gp120 may form toxic inclusions in lysosomes or other organelles which lead to cell death. For instance, gp120 accumulation inside lysosomes could disrupt the metabolism of sphingolipids and produce toxic bio-products, such as ceramide, as described by other investigators [89].

### 4.2. gp120 and the Neuronal Cytoskeleton

One of the immediate consequences of gp120 endocytosis is binding to the neuronal cytoskeleton (Figure 3). The neuronal cytoskeleton is crucial for the establishment and maintenance of spatial organization and architectural support of axons and dendrites. In addition, the cytoskeleton is crucial to preserve the functionality of intracellular transport and dendritic spines. The neuronal cytoskeleton is made of microtubules, actin, neurofilaments and their associated proteins. One of the components of neuronal microtubules is class III β tubulin (TUBB3). The C-terminal tail domain (CTT) of TUBB3 has a binding site for an α-helix motif of gp120 [90]. This binding occurs at nanomolar concentrations of gp120, suggesting that the binding of gp120 to the cytoskeleton is in a high-affinity range. This binding leads to several changes in cytoskeletal components and in particular to posttranslational modification of tubulin. In fact, it has been shown that gp120 reduces microtubule (MT) polymerization and acetylation of tubulin [73]. Reduced levels of tubulin acetylation cause axonal transport defects, as seen in various neurological diseases, such as Huntington’s disease [91] and Charcot–Marie–Tooth disease [92]. These defects can be reversed by restoring tubulin acetylation levels [93].

Tubulin acetylation may decrease the binding of microtubule-associated proteins, such as Tau and microtubule associated protein 2, which, in turn, regulates the stabilization of MTs [94,95]. Moreover, gp120 binding to the CTT of TUBB3 could provide steric interference with the binding domain of motor proteins, such as kinesin-1 and dynein/dynactin and tubulin [96]. In fact, acetylation at Lys 40 of tubulin plays a role in recruiting molecular motors [91,97]. These motor proteins play a crucial role in axonal trafficking of organelles, including mitochondria, synaptic vesicles and trophic factor-containing vesicles, including brain-derived neurotrophic factor (BDNF). In fact, it has been demonstrated that gp120 interferes with the axonal transport mediated by kinesins and dynein [98]. The impairment of axonal transport explains why various degrees of axonal pathology are seen in HAND [4,99]. This phenomenon is not unique to HAND but appears to be seen also in Huntington’s disease, in which mutated huntingtin inhibits axonal transport by acting at kinesin-1 motor protein [100] or by interacting with mitochondrial fission GTPase, which regulates mitochondrial transport [101].

Binding of gp120 to tubulin and the subsequent alteration of motor protein association with MTs are attractive mechanisms to explain this type of gp120′s toxicity because Helix-A, a peptide that displaces gp120 and prevents it from binding to TUBB3, prevents gp120-mediated neuronal simplification and neurotoxicity [90]. The pathology of the axonal cytoskeleton is a new mechanism to explain the loss of synapses seen in the brain of patients with HAND. Importantly, the binding of gp120 to TUBB3 and the fact that this binding can be reversed is an appealing target for the development of a future adjunct therapy.

### 4.3. gp120 and Axonal Transport

There are other potential mechanisms that could explain the link between binding to microtubules and neurotoxicity. One of the immediate consequences of gp120 impairment of axonal transport may be the decreased movement of mitochondria. Neurons are highly dependent on these essential organelles because they control high-energy intermediates, including adenosine triphosphate (ATP), which is in high demand in neurons. ATP is required at synapses for synaptic vesicular release and recycling [102]. Moreover, mitochondria has several functions crucial for neuronal maintenance and survival, including spine maturation, regulation of Ca^2+^ homeostasis [103] and production of reactive oxygen species [104]. In order to maintain energy homeostasis and to continue essential activities, neurons must precisely establish an adequate distribution of mitochondria to axons and dendrites through the process of fusion and fission [105,106]. Fusion is the process of exchanging mitochondrial DNA and other components, with the goal of repairing damaged mitochondria. The opposite process, fission, is required for mitochondrial transport. Both processes utilize various proteins, such as mitofusins, and GTPase dynamin-related protein (DRP), located on the outer membrane of mitochondria, and optic atrophy protein 1, located in the inner membrane. Further, those mitochondria that accumulate defective proteins or DNA must be either repaired by fusion with healthy mitochondria or cleared from the cell by a selective process of mitophagy [107]. For these processes, axonal transport is essential because mitochondria need to be retrogradely transported to the cell body by MTs to be repaired or degraded.

Mitochondrial impairment, and alteration in the distribution and function of mitochondria in particular, have been described in several animal models of HAND, including gp120 transgenic mice [108], Tat transgenic mice [109] and HIV transgenic rats [72]. These experimental data are important for a better understanding of HAND neuropathology because mitochondria in the brain of HAND patients exhibit impaired mitochondrial fission and distribution [110]. This suggests that HIV, through its viral proteins, is neurotoxic, exerting these effects by altering the axonal transport of mitochondria.

## 5. gp120 and the p75^NTR^

The above considerations describe processes by which HIV could reduce the integrity of axons. However, HAND also exhibits loss of dendritic synapses. Synapto-dendritic injury can also be caused by a lack of neurotrophic factors [111]. This issue is relevant because it has been reported that HAND subjects display lower BDNF levels [112] compared to HIV-positive subjects with normal cognitive function. BDNF is a neurotrophin that plays a key role in dendritic branching and spine morphology [113,114], neurogenesis [115] and the survival of synapses in both animals [116] and humans [117]. Moreover, clinical studies have shown an association between low levels of plasma BDNF and a decrease in cognitive functions [118]. Thus, a reduced availability of BDNF may be one of the molecular mechanisms leading to synaptic simplification in HIV-positive subjects, which leads to cognitive impairment in HAND.

There are many mechanisms that could be utilized by HIV to decrease BDNF. Elucidation of these mechanisms may shed light on potential causes for HAND. BDNF, as for other neurotrophins, is synthesized as a large, glycosylated precursor (proBDNF). In the Golgi apparatus, proBDNF is proteolitically cleaved at the Arg-*X*-Lys/Arg-Arg site to mature BDNF (mBDNF) by furin [119], an endoprotease that has been shown to cleave several precursor proteins. In addition, proBDNF can be cleaved by other proconvertases within synaptic vesicles [120]. When proBDNF is not cleaved, it mediates biological effects that are opposite to those elicited by mBDNF, including neuronal apoptosis [121] and presynaptic terminal retraction [122]. The opposite biological effect of mBDNF and pro-BDNF are mediated by two different receptors, the protooncogene TrkB and the p75 neurotrophin receptor (p75^NTR^), a member of the tumor necrosis factor family of receptors. BDNF binds selectively to TrkB whereas the pro-neurotrophins bind with a similar affinity to p75^NTR^ [123]. The pro-domain interferes with TrkB receptor binding and activation and thus renders pro-neurotrophins selective p75^NTR^ ligands. High affinity of the proneurotrophins is conferred by the pro-domain interaction with sortilin, a sorting receptor, which associates with p75^NTR^ to bind pro-neurotrophins and promote cell death [124].

The proconvertase furin, which is important for the initial infection of HIV by cleaving the glycoprotein gp160 into gp120 and gp41 [125], is decreased in the brain of HAND subjects [112]. The decrease in furin levels is mediated by gp120 because the viral protein decreases the levels of furin in neuronal cultures [112] as well as in vivo [126]. Consequently, HAND brains as well as neuronal cultures exposed to gp120 exhibit an increase in proBDNF [112]. The net outcome of gp120 interference with proBDNF processing is that more proBDNF and less mBDNF proteins are available to synapses. This pathological environment favors a proBDNF/p75^NTR^ activation, which triggers the loss of synapses and dendritic spines (Figure 3). Indeed, when rat primary cortical neurons are exposed to gp120, they release proBDNF, whose neurotoxic effect is inhibited by tat-Pep5, a cell-permeable p75^NTR^ antagonist, or a p75^NTR^ blocking antibody [112]. Further, primary cortical neurons obtained from p75^NTR-/-^ rodents are protected against apoptosis following gp120-induced proBDNF release [127]. Likewise, the loss of hippocampal dendritic spines and synapse integrity observed in gp120tg mice is completely rescued in gp120tg rodents lacking one p75^NTR^ allele [127]. Moreover, p75^NTR^ activates c-Jun N-terminal kinase or JNK [128] and inhibits anti-apoptotic proteins Bcl-2 or Bcl-x_L_ [129], which have both been implicated in gp120-mediated cell death [130,131]. Thus, it is plausible to suggest a scenario by which HIV decreases the processing of proBDNF to mBDNF, which will initiate the processing of dendritic degeneration (Figure 3). Thus, future research should aim at developing new pharmacological agents that are able to inhibit proBDNF/p75^NTR^ activity and therefore be used against HIV-mediated neuronal toxicity.

## 6. Conclusions and Future Directions

It is curious that the molecular events leading to the neuropathological features of HAND remain elusive despite several years of investigation of a disease with such profound abnormalities in cognitive function. Even the timing of synaptic loss is uncertain. While subtle, this loss could arise from a chronic presence of viral proteins in the CNS, a process that could initiate as soon as the virus enters the brain. As the CNS becomes a sanctuary of HIV infection despite the use of cART, a cure for HAND is of paramount importance. Finding a cure requires more detailed mechanisms leading to HAND pathology. This is important because, although the pharmacological approach for infection is our best chance to reduce the severity of HAND, it may not be suited to cure the neuropathology leading to HAND. Biological therapies could be an alternative because they use physiological compounds used by the organism to promote synapses or, at least, foster synaptic plasticity. However, these therapies presuppose some knowledge of pathogenic mechanisms and address specific steps in the pathogenesis cascade.

Experimental data suggest that HIV, through gp120, causes an initial “traumatic” event in axons and dendrites that may culminate in retrograde degeneration and synaptic simplifications. HAND brains exhibit a similar scenario. Thus, the pathology of axons observed in HAND could be link to the ability of gp120 to alter the efficient bidirectional axonal transport that is crucial for neuronal survival.

Deacetylation of TUBB3 appears to be a crucial mechanism leading to the pathology of axons and dendrites observed in HAND. Deacetylation of TUBB3 is mainly mediated by histone deacetylase 6 (HDAC6) [132,133]. HDAC6 acts primarily in the cytoplasm and has a variety of targets [132]. These targets regulate several processes within cells, including cell signaling and cytoskeletal components [134], which all contribute to neuronal survival. For example, HDAC6 deacetylates nine lysine residues on cortactin, a protein that can promote the rearrangement and polymerization of the actin cytoskeleton. Maintaining the acetylation of cortactin promotes postsynaptic density protein 95 (PSD95) clustering [135], which is an essential mechanism for the formation of functional dendritic spines and regulation of excitatory synapses. HDAC6 inhibition has been found to protect rat cortical neurons against gp120-mediated loss of synapses. Thus, HDAC6 could be a valid therapy for HAND. Two compounds known to inhibit HDAC6 have shown some promising properties against gp120 neurotoxicity [98], namely, tubacin and Ricolinostat. The latter has been used orally as a therapy for multiple myeloma [136] and other forms of cancer [137]. Thus, a major part of a future task will be the devotion of more attention to the development of unconventional therapies for HAND that target not only HIV but also the molecular mechanisms utilized by the virus to promote neurodegeneration. Each approach has its unique strengths and limitations. It is our hope that the mechanisms presented in this article will assist investigators in understanding how the initial infection contributes to the clinical presentation of HAND and, most importantly, guide them to work on developing therapeutic strategies to minimize neuronal degeneration in HAND patients.

## Data Availability

Not applicable.
